# Puerarin Enhances Eggshell Quality by Mitigating Uterine Senescence in Late-Phase Laying Breeder Hens

**DOI:** 10.3390/antiox14080960

**Published:** 2025-08-05

**Authors:** Zhenwu Huang, Guangju Wang, Mengjie Xu, Yanru Shi, Jinghai Feng, Minhong Zhang, Chunmei Li

**Affiliations:** 1 College of Animal Science and Technology, Nanjing Agricultural University, Nanjing 210095, China; 2019105026@njau.edu.cn; 2 State Key Laboratory of Animal Nutrition and Feeding, Institute of Animal Sciences, Chinese Academy of Agricultural Sciences, Haidian, Beijing 100193, China; guangju.wang@wur.nl (G.W.); xumengj13@163.com (M.X.); shiyanru@caas.cn (Y.S.); fengjinghai@caas.cn (J.F.)

**Keywords:** puerarin, antioxidant, aged breeder hen, uterus, calcium transport

## Abstract

The deterioration of uterine calcium transport capacity induced by aging is a common problem for late-laying period hens, causing decline in eggshell quality. This study aimed to investigate the effects and possible regulatory mechanisms of dietary puerarin (PU) on calcium transport and eggshell quality in aged hens. Two hundred eighty-eight Hubbard Efficiency Plus broiler breeder hens (50-week-old) were randomly allocated to three dietary treatments containing 0, 40, or 200 mg/kg puerarin (PU), with 8 replicates of 12 birds each, for an 8-week trial. The results demonstrated that dietary PU ameliorated the eggshell thickness and strength, which in turn reduced the broken egg rate (*p* < 0.05). Histological analysis showed that PU improved uterus morphology and increased epithelium height in the uterus (*p* < 0.05). Antioxidative capacity was significantly improved via upregulation of *Nrf2*, *HO-1*, and *GPX1* mRNA expression in the uterus (*p* < 0.05), along with enhanced total antioxidant capacity (T-AOC) and glutathione peroxidase (GSH-PX) activity, and decreased levels of the oxidative stress marker malondialdehyde (MDA) (*p* < 0.05). Meanwhile, PU treatment reduced the apoptotic index of the uterus, followed by a significant decrease in expression of pro-apoptotic genes *Caspase3* and *BAX* and the rate of *BAX*/*BCL-2*. Additionally, calcium content in serum and uterus, as well as the activity of Ca^2+^-ATPase in the duodenum and uterus, were increased by dietary PU (*p* < 0.05). The genes involved in calcium transport including *ERα*, *KCNA1*, *CABP-28K*, and *OPN* in the uterus were upregulated by PU supplementation (*p* < 0.05). The 16S rRNA gene sequencing revealed that dietary PU supplementation could reverse the age-related decline in the relative abundance of *Bacteroidota* within the uterus (*p* < 0.05). Overall, dietary PU can improve eggshell quality and calcium transport through enhanced antioxidative defenses and mitigation of age-related uterine degeneration.

## 1. Introduction

The eggshell, a vital bioceramic container, protects the chick embryos against physical damage and microbial contamination, and its quality has great biological and economic significance for the poultry industry [1,2]. However, the quality of eggshell deteriorates rapidly with age in older broiler breeders, which have detrimental effects on breeding egg collection, transport, and hatchability [3]. It has been reported that about 12–20% of eggs suffer from eggshell quality problems in the late laying period, which results in a substantial economic loss [4]. The chicken eggshell mainly consists of 95% calcium carbonate [1,5]. Thus, calcium is one of the vital nutritional factors critical for the formation of eggshells in hens. The small intestine is generally a major site of calcium absorption in poultry, especially in the duodenum [6]. Generally, most of the absorbed calcium ions are transported through the blood circulation to the hen’s uterus (eggshell gland) and used in the formation of eggshells [7]. Calcium-binding protein, along with plasma membrane Ca^2+^-ATPase, serves as a crucial mediator in the absorption and translocation of calcium ions, playing a pivotal role in the biological process of eggshell formation [8]. Calbindin-D28K (*CABP-28K*), mainly expressed in the tubular gland cells of the uterus, is an important calcium-binding protein in hens involved in calcium transport [9]. Thereby, the expression level of *CaBP-28K* and Ca^2+^-ATPase activity in the uterus of aging hens plays a critical role in the quality of the eggshell.

Normally, tissues and organs of young hens exist in a state of equilibrium between oxidative processes and antioxidant defenses [10]. However, prolonged high-frequency egg production can increase the accumulation of reactive oxygen species (ROS) in aged hens, leading to exacerbated cellular damage and apoptosis [11]. This typically manifests itself macroscopically as histomorphological damage, as well as organ senescence and functional decline [12]. Substantial evidence suggests that the deterioration in eggshell quality associated with aging is closely linked to oxidative damage in reproductive organs [13,14]. Exacerbated aging could weaken the calcium transportation of aged hens through decreased activity of Ca^2+^-ATPase [2], which is one of the main causes of reduced eggshell quality. Additionally, it is worth noting that the hen’s uterus is not a sterile environment and is connected to the gut via the cloaca. Recent evidence indicates that uterine microbiota metabolites play an important role in regulating the cell cycle and apoptosis within the uterus [15]. With aging, the uterine microbiota undergoes significant shifts in hens, and the microbiota dysbiosis may further exacerbate physiological changes in the aged uterus [15,16]. The findings of Lu et al. revealed that changes in the microbial composition (e.g., the phyla *Proteobacteria*, *Firmicutes*, and *Bacteroidetes*) of the uterus were closely linked to improvements in egg quality and uterine tissue health [16]. Particularly, *Bacteroides* showed a positive correlation with the effective thickness and the concentrations of K^+^, Na^+^, and S^2−^ in the eggshell [16]. This suggests that uterine microbes have a crucial role in the physiological function of the shell gland. Therefore, the relationship between age-related changes in the uterine microbiota and impaired eggshell quality warrants further investigation.

Supplementation with natural antioxidants is thought to protect aging tissues from excessive oxidative damage in aged hens [17]. Substantial evidence has indicated that dietary supplements with phytoflavonoids could improve eggshell quality in aged hens [2,18,19]. Puerarin (PU), an isoflavone glycoside, is the main active substance of the root in *Pueraria lobata* (*Willd.*) and has a variety of biological activities, including anti-inflammatory, antioxidant, anti-aging, and antibacterial [20]. Due to its similar structure to estrogen, puerarin also exhibits weak estrogen-like activity in animals [21]. A recent study reported that puerarin (200 mg/kg) can increase the antioxidant capacity and improve intestinal morphology in aged laying hens [22]. Furthermore, studies also indicated that PU could alleviate oxidized oil-induced oxidative and inflammatory injury by activating the *Nrf2* signaling pathways in chicks [23]. By modulating microbial community structures, puerarin could also suppress Salmonella Enteritidis-induced gut microbiota dysbiosis in chicks [24]. However, the effects and regulatory mechanisms of dietary puerarin on the eggshell quality in aged broiler hens remain unclear. We hypothesized that dietary puerarin supplementation would improve eggshell quality by enhancing calcium transport, modulating antioxidant pathways, and altering uterine microbiota in aged hens. Therefore, this study aimed to evaluate the effects of dietary puerarin supplementation on antioxidant capacity, calcium transport, and uterine microbiota in aged breeder hens, to elucidate mechanisms underlying improved eggshell quality.

## 2. Materials and Methods

### 2.1. Animals and Experimental Design

A total of 288 Hubbard Efficiency Plus broiler breeder hens (50-week-old) were randomized into three groups (CON, LPU, and HPU) supplemented with different puerarin doses (0, 40, and 200 mg/kg). There were 8 replicates per treatment, with 12 hens per replicate. The control treatment was fed with a basic corn-soybean diet, referring to the nutrient requirements of the China National Feeding Standard of Chicken (NY/T33-2004) [25], and is shown in Table 1. Puerarin monomer, with a purity of 98%, was purchased from Tianfeng Bio Limited (Xi’an, China). All hens were raised on the Jiulian 10th Breeding Farm (Qingdao, China). Each set of six adjacent cages formed one replicate, with different replicates separated by baffles. A one-week acclimation period was implemented before the eight-week formal experiment to adjust egg production rates across replicates. During the trial, each hen was fed 146 g per day at 4:30 am. Water was provided ad libitum via nipple drinkers. Lighting was provided by both natural and artificial sources, with a daily photoperiod of 16 h. In addition, the temperature and humidity in the breeding farm room were 20–23 °C and 50–60%, respectively.

### 2.2. Misshapen Egg Rate, Broken Egg Rate, and Eggshell Quality

The number of broken and misshapen eggs laid per repetition was measured on a daily basis in order to assess the incidence of broken and misshapen eggs. At the 4th and 8th weeks of the experiment, 16 eggs from each treatment (two eggs per replicate) were randomly collected and immediately measured for egg quality. The egg shape index was measured using the FHK egg shape determinator (Fujihira Industry Co., Ltd., Tokyo, Japan). Eggshell strength was measured using the egg force reader (EFR-01; Orka, Ramat HaSharon, Israel). After separating the egg yolk and egg white, the weight of the eggshell was measured by an electronic balance. The eggshell portion was calculated by dividing the eggshell weight by the egg weight. Finally, eggshell thickness was measured by a vernier caliper (Mitutoyo, S-530, Kawasaki, Japan) with the blunt end, middle, and sharp end.

### 2.3. Sample Collection

At the end of the official experiment, one hen per replicate was randomly selected for sample collection at 9:00 am. The blood sample was collected from the wing vein of these hens. The serum was separated by centrifugation of blood (3000× *g*, 15 min, 4 °C) and stored at −80 °C for future biochemical analysis. Hens were euthanized by cervical dislocation for immediate sampling of the duodenum and uterus for weighing. Approximately 2 g of tissue samples were promptly frozen in liquid nitrogen and stored at −80 °C. Uterine microbial samples were collected using sterile cotton swabs and were processed according to the method of Dai et al. [15]. Subsequently, part of the uterus was fixed in 4% paraformaldehyde for histological analyses.

### 2.4. Analysis of Calcium Content, Phosphorus Content, and Ca^2+^-ATPase

About 200 mg of frozen duodenum or uterus tissue was placed in 1 mL of physiological saline. After intense homogenization, the supernatant was used to determine protein concentration using the BCA Protein Quantification Kit (P0010S, Beyotime Biotechnology, Shanghai, China). Subsequently, the commercial kits (C004-2-1, C006-1-1, and A070-4, Jiancheng Bioengineering Institute, Nanjing, China) were used to detect the calcium and phosphorus levels and the activity of Ca^2+^-ATPase in aged hens. Serum samples were diluted in a 1:5 ratio for calcium and phosphorus measurement. Ca^2+^-ATPase activity and mineral levels were normalized to protein content.

### 2.5. Morphology Analysis of the Uterus

The fixed tissues were dehydrated by ethanol and xylene and then embedded in paraffin. Subsequently, tissue sections (5 μm) were subjected to RM2235 rotary microtome (Leica Microsystems, Wetzlar, Germany). The sections were dewaxed in xylene, rehydrated through a graded ethanol series, and stained with hematoxylin–eosin (H&E) using a commercial staining kit (Servicebio, Wuhan, China). The images of the slices were taken using a DM68 Leica bright-field/fluorescence microscope (Leica, Wetzlar, Germany). Five random visual fields in sections of hens were selected and analyzed using the Image-Pro Plus software 6.0 (Media Cybernetics, Washington, DC, USA). Uterine parameters were measured according to our previous methods [2], including villus length, mucosal fold width, and epithelial cell height.

### 2.6. Antioxidation-Level Analysis of the Uterus

The supernatant from uterine tissues was extracted using RIPA Lysis Buffer, and total protein was quantified using an Enhanced BCA Protein Assay kit (P0013B and P0010S, Beyotime Biotechnology, Shanghai, China). Total antioxidant capacity (T-AOC), glutathione peroxidase (GSH-PX), total superoxide dismutase (T-SOD) activity, and malondialdehyde (MDA) levels were determined using the commercial kits (A0015-2-1, A005-1, A001-1, and A003-1-2, Jiancheng Bioengineering Institute, Nanjing, China). All assays were performed according to the manufacturers’ instructions, and values were normalized to protein concentration.

### 2.7. TUNEL Assay of the Uterus

The uterine cell apoptosis was detected using the TUNEL BrightRed Apoptosis Detection Kit (A113-3, Vazyme Biotech, Nanjing, China). Paraffin-embedded sections were deparaffinized and rehydrated through a graded ethanol series, then treated with 20 μg/mL proteinase K (room temperature, 20 min). After washing in PBS, sections were incubated with TdT enzyme and BrightRed Labeling Mix at 37 °C for 1 h in the dark. Later, sections were stained with DAPI (C1005, Beyotime Biotechnology, Shanghai, China) for 5 min in the dark. The fluorescent images were acquired using a DM68 Leica bright-field/fluorescence microscope (Leica, Wetzlar, Germany). The Image J software (Media Cybernetics, Washington, DC, USA) was used for image analysis and processing. Apoptosis ratio was taken as apoptotic cells (red)/total cells (blue) × 100%.

### 2.8. Total RNA Extraction and Real-Time Quantitative PCR

Total RNA was extracted from the uterus of hens by using the Total RNA Extraction Kit (ER206-02, I-Presci Scientific, Beijing, China). The concentration, purity, and integrity of the RNA were detected by a micro-spectrophotometer (Bio-Rad, Hercules, CA, USA). A total of 1000 ng RNA was reverse transcribed using a reverse transcription kit (R323-01, Vazyme Co., Nanjing, China). Subsequently, qRT-PCR was conducted using a real-time PCR machine (Quant Studio7 Flex, Thermo Fisher Scientific, Waltham, MA, USA) and ChamQTM SYBR qPCR Master Mix (Q311-02/03, Vazyme Co., Nanjing, China). All amplification dissolution curves had single peaks and revealed no nonspecific amplification. The primer sequences were synthesized by Sangon Biotechnology (Shanghai, China) and provided in Table S1. The relative expression of mRNA was calculated by the 2^^(−∆∆Ct)^ method normalized to ACTB.

### 2.9. Sequencing of the 16S Ribosomal RNA (rRNA) Gene

The 0 and 200 mg/kg PU treatment groups were selected to detect microbiological changes in the shell gland based on eggshell quality and biochemical indicators. Ten microbial samples in the uterus of hens were randomly selected (five per group) for 16S rRNA gene sequencing. Genomic DNA was extracted using the CATB method, and the 16S V3-V4 regions were amplified using specific primers (341F, CCTAYGGGRBGCASCAG; 806R, GGACTACNNGGGTATCTAAT). PCR reactions used Phusion High-Fidelity PCR Master Mix (New England BioLabs, Ipswich, MA, USA). Libraries were prepared with the NEBNext Ultra II DNA Library Prep Kit (New England BioLabs), and sequencing was performed on an Illumina NovaSeq 6000 platform (San Diego, CA, USA). All steps were conducted by Beijing Novogene Technology Co. (Beijing, China). The specific steps of visualization and analysis were referred to the methods of earlier literature [26]. All analyses were executed on Novogene Cloud Platform (https://magic.novogene.com, accessed on 11 July 2025).

### 2.10. Statistical Analysis

All data were analyzed using SPSS (version 26.0, SPSS Inc., Chicago, IL, USA). Eggshell quality, biochemical indicators, morphological analysis, and gene expression data were analyzed by one-way analysis of variance (ANOVA), and Duncan’s multiple-range tests were used to determine significant differences. 16S rRNA gene sequencing data were analyzed by independent-samples T tests. Spearman’s rank correlation analysis was performed to explore the potential relationship between uterine microbiota (genus level) and apoptosis-related genes in the uterus. Results were represented as mean ± standard error of the mean (SEM), with statistical significance defined at *p* < 0.05.

## 3. Results

### 3.1. Misshapen Egg Rate and Broken Egg Rate

To assess the effect of PU supplementation on reducing the unqualified egg rate of aged breeder hens, we analyzed the broken and misshapen egg rate throughout the experimental period (Table 2). Compared with the CON group, dietary supplementation with 200 mg/kg PU resulted in a significant reduction in the broken egg rate between weeks 5–8 and weeks 1–8 (*p* < 0.05). The misshapen egg rate tended to decrease with increasing PU dose (*p* = 0.084).

### 3.2. Eggshell Quality

To assess the changes in eggshell quality of the late-laying period broiler breeder hens, we examined the eggshell quality among three groups at weeks 4 and 8 (Table 3). Of note, shell strength tended to increase with PU supplementation at weeks 4 and 8 (*p* = 0.053 and 0.078). Similarly, shell thickness showed an increasing trend with PU supplementation at week 4 (*p* = 0.094). At week 8, the shell thickness was significantly higher in the 200 mg/kg PU group than in the CON group (*p* < 0.05). However, PU treatments had no significant effect on eggshell weight at week 4 or 8.

### 3.3. Calcium Content, Phosphorus Content, and Activity of Ca^2+^-ATPase

**Figure 1:**
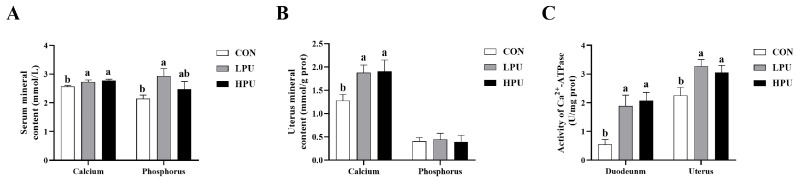
Effects of dietary puerarin on calcium content, phosphorus content, and Ca^2+^-ATPase enzyme activity of aged hens. (**A**) Calcium and phosphorus levels in serum, (**B**) Calcium and phosphorus levels in uterus, (**C**) Ca^2+^-ATPase enzyme activity in duodenum and uterine. Mean value ± SEM is used to represent data (n = 8). a, b Different letters represent statistically significant differences among the groups (*p* < 0.05).

As represented in Figure 1, dietary supplementation with PU significantly increased serum and uterus calcium content compared with the CON group (*p* < 0.05, Figure 1A,B). Similarly, the serum phosphorus content in the LPU group was significantly elevated compared to the CON group (*p* < 0.05, Figure 1A), whereas uterine phosphorus showed no difference among groups (Figure 1B). Additionally, dietary 40 or 200 mg/kg PU enhanced Ca^2+^-ATPase activity in the duodenum and uterus (*p* < 0.05, Figure 1C).

### 3.4. Histomorphology of the Uterus

**Figure 2:**
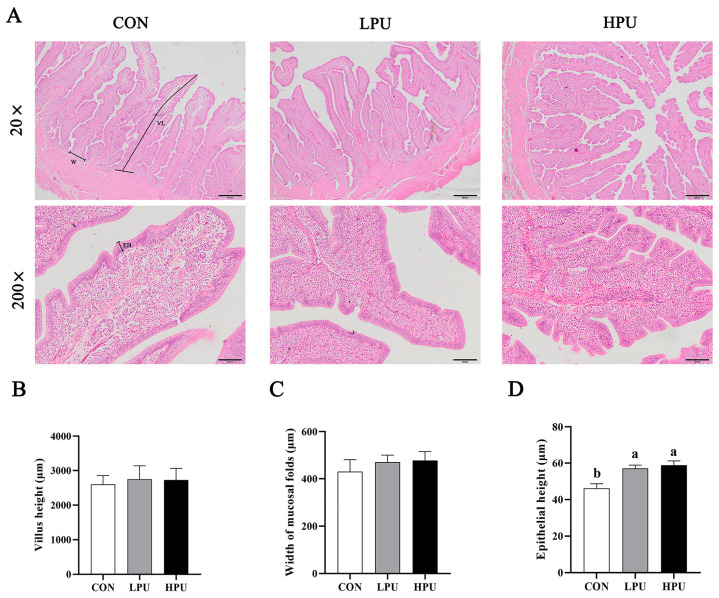
Effects of dietary puerarin on morphological analysis of uterus in aged hens. (**A**) Representative image of HE staining for uterus. (**B**) Villus length, (**C**) Width of mucosal folds, (**D**) Epithelial height. (**A**): Villus length (VL), Width of mucosal folds (W), Epithelial height (EH). Mean value ± SEM is used to represent data (n = 8). a, b Different letters represent statistically significant differences among the groups (*p* < 0.05).

HE staining was used to observe the morphology of the uterus in aged hens (Figure 2A). We recorded the changes in villus length, width of mucosal folds, and epithelial height induced by dietary PU supplementation. Interestingly, dietary supplementation with 40 and 200 mg/kg PU significantly increased epithelium height compared with the CON group (*p* < 0.05, Figure 2D), whereas PU inclusion did not affect villi length or mucosal fold width (*p* > 0.05, Figure 2B,C). Additionally, it is noted that PU reduced the loose connective tissue and increased the glandular density of the uterus.

### 3.5. Oxidative Status of the Uterus

**Figure 3:**
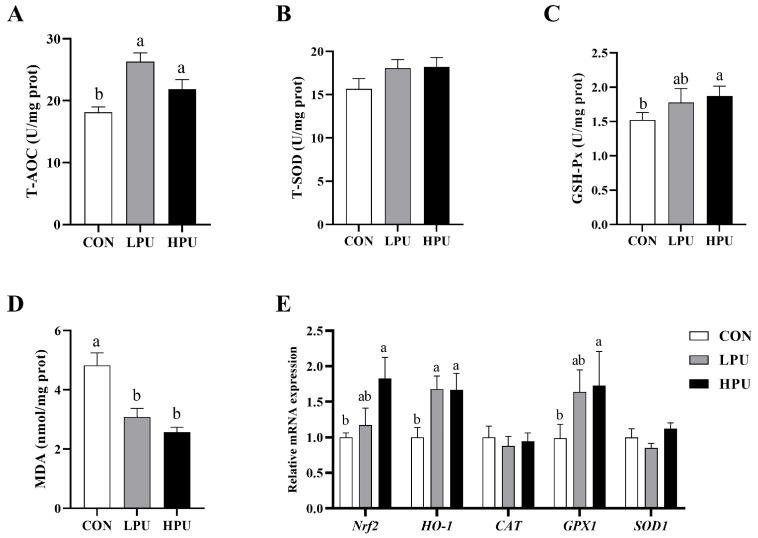
Effects of dietary puerarin on the oxidative status in the uterus of aged breeder hens. (**A**) Activity of T-AOC. (**B**) Activity of T-SOD. (**C**) Activity of GSH-Px. (**D**) MDA content. (**E**) The relative mRNA expression levels of genes related to antioxidants. a, b Different letters represent statistically significant differences among the groups (*p* < 0.05).

Figure 3 shows the effects of dietary PU on uterine oxidative status in aged breeder hens. Compared with the CON group, supplementation with 40 and 200 mg/kg PU significantly increased the levels of T-AOC and GSH-Px in the uterus (*p* < 0.05, Figure 3A,C). Additionally, MDA content in the uterus was significantly reduced (*p* < 0.05, Figure 3D), whereas no significant differences were observed in T-SOD activity among the three groups (Figure 3B). The expression levels of anti-oxidative-related genes in the uterus are shown in Figure 3E. Dietary supplementation with 200 mg/kg PU significantly upregulated the mRNA expressions of *Nrf2* and *GPX1* (*p* < 0.05). In addition, both 40 and 200 mg/kg PU treatment significantly increased the expression of *HO-1* (*p* < 0.05). In contrast, mRNA expression of *CAT* and *SOD1* remained unaffected by PU supplementation.

### 3.6. Apoptosis of the Uterus

**Figure 4:**
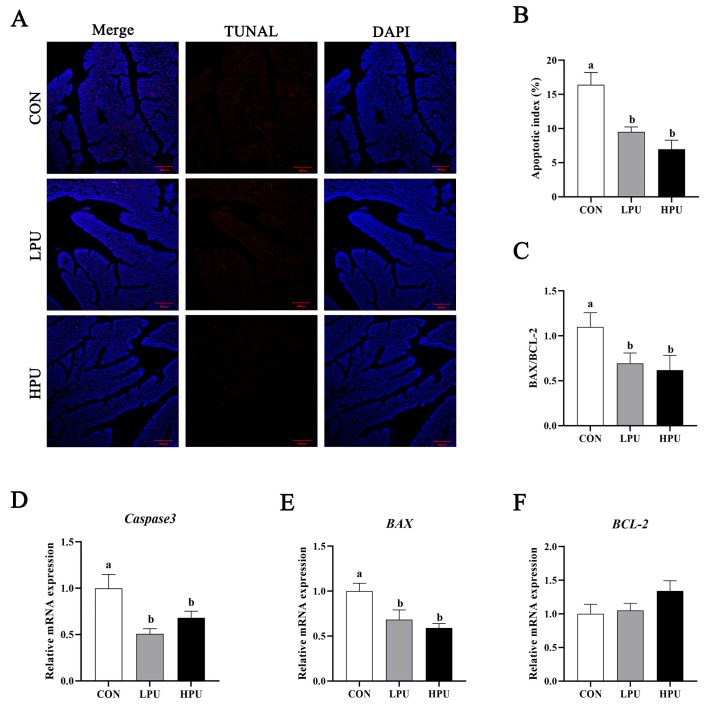
Effects of dietary puerarin on apoptosis of uterus in aged breeder hens. (**A**) TUNEL assay of the uterine. (**B**) Statistical analysis of apoptotic index. (**C**) The ratio of *BAX* to *BCL-2*. (**D**–**F**) The relative mRNA expression levels of *Caspase3*, *BAX*, and *BCL-2*. a, b Different letters represent statistically significant differences among the groups (*p* < 0.05).

To evaluate the effect of PU supplementation on uterine apoptosis, we performed TUNEL staining and quantified apoptosis-related gene expression (Figure 4). Uterine apoptosis was significantly alleviated by dietary PU (Figure 4A). Apoptotic activity was quantified via the apoptotic index (percentage of apoptotic glandular cells per tissue). We found that dietary supplementation with 40 and 200 mg/kg PU significantly decreased the apoptotic index compared with the CON group (*p* < 0.05, Figure 4B). Consistently, PU supplementation significantly decreased mRNA expressions of *Caspase3* and *BAX* (*p* < 0.05, Figure 4D,E). Furthermore, the *BAX*/BCL2 ratio significantly decreased in the LPU and HPU group (*p* < 0.05, Figure 4C).

### 3.7. Calcium Transportation Gene Expression in the Uterus

**Figure 5:**
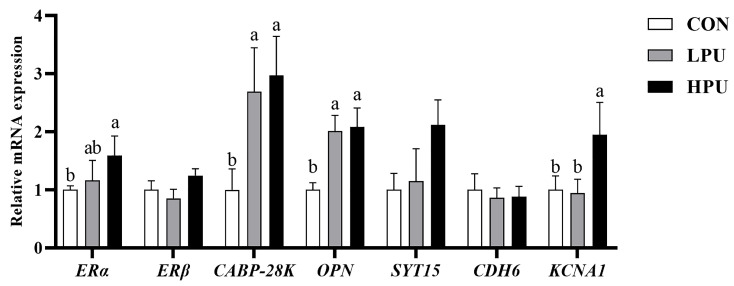
Effects of dietary puerarin on the relative mRNA expression of calcium transportation in uterine of aged hens. a, b Different letters represent statistically significant differences among the groups (*p* < 0.05).

As shown in Figure 5, dietary supplementation with 200 mg/kg PU significantly up-regulated the mRNA expressions of *ERα* and *KCNA1* compared with the control group (*p* < 0.05). In addition, 40 and 200 mg/kg PU supplementation remarkably increased *CABP-28K* and *OPN* expression (*p* < 0.05). However, the mRNA expressions of *ERβ*, *CDH6*, and *SYT15* showed no significant difference among groups (*p* > 0.05).

### 3.8. Structure of the Uterine Microbiota

**Figure 6:**
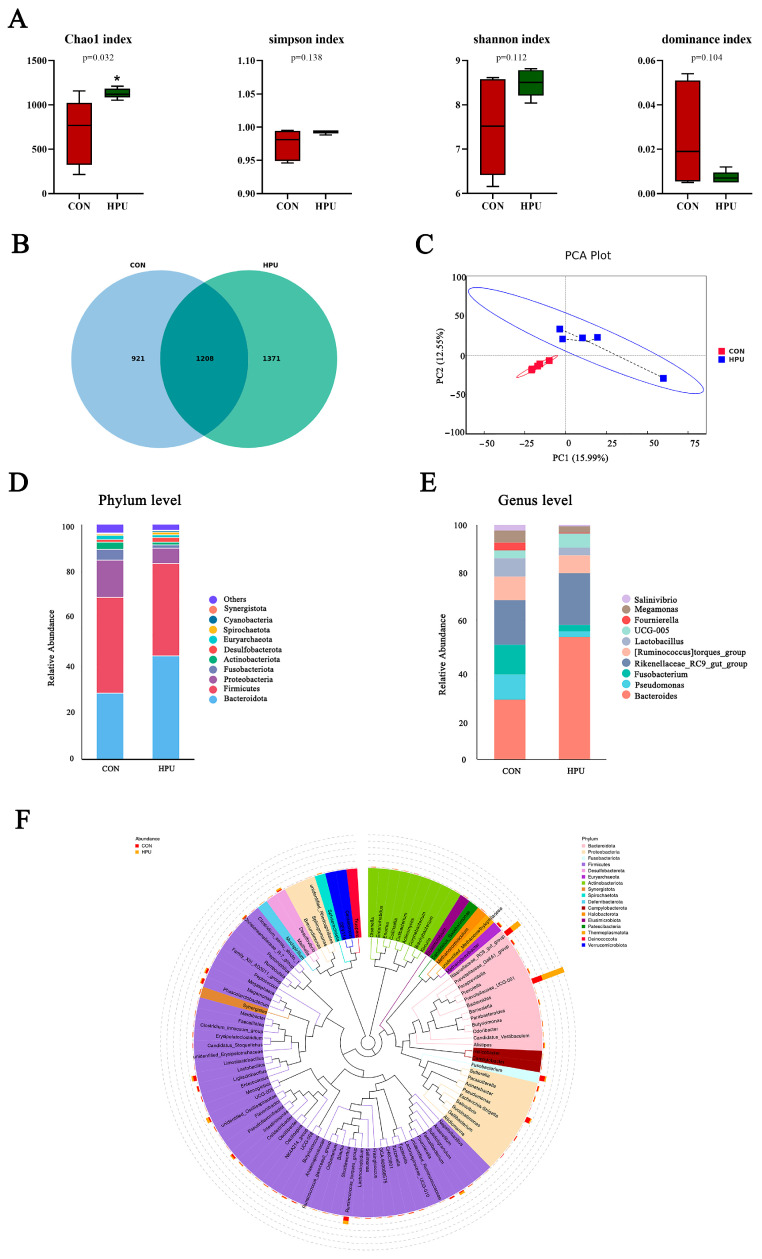
Effect of dietary puerarin on uterine microbial communities. (**A**) Alpha diversity analysis. (**B**) Venn diagram of OTU level. (**C**) Principal component analysis (PCA) scores plot of the samples. (**D**,**E**) Relative abundance of the bacteria at the phylum and genus. (**F**) Enriched taxa represented in cladograms. Mean value ± SEM is used to represent data (n = 5). Asterisks indicate significant differences between groups (*p* < 0.05).

Alpha and beta diversity analyses were used to characterize microbiota richness and diversity between the CON and HPU groups (Figure 6). Compared with the CON group, the HPU group had significantly increased Chao1 index values (*p* < 0.05, Figure 6A). For Simpson, Shannon, and dominance indices, no significant differences were observed between groups (*p* > 0.05). Venn diagram analysis revealed 1208 shared OTUs, with 921 unique to CON and 1371 unique to HPU (Figure 6B). Beta diversity analysis was presented by principal component analysis (PCA) (Figure 6C), showing clear microbial separation between groups.

**Figure 7:**
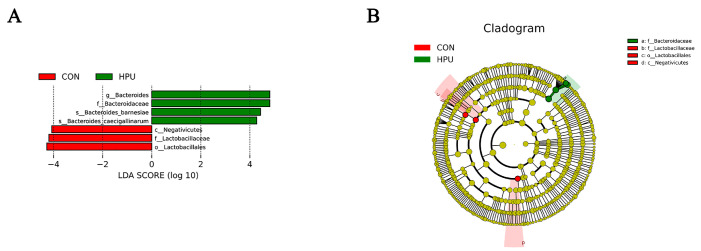
Different structures of uterine microbiota in the CON and HPU groups according to the LEfSe analysis. (**A**) Linear discriminant analysis (LDA) score distribution. (**B**) Cladogram plot of the biomarkers. The score of LDA > 4 and α =0.05.

The relative abundance of the microbial community in the uterus was analyzed at the phylum and genus levels (Figure 6D,E). At the phylum level, PU treatments tended to increase the relative abundance of *Bacteroidota* in the uterus (*p* = 0.056) (Table 4). At the genus level, *Bacteroides* abundance was significantly increased in the HPU group (*p* < 0.05). Cladogram analysis highlighted enriched taxa in the uterine microbiota (Figure 6F). Further, LEfSe analysis identified distinctive bacteria between groups (*p* < 0.05, LDA score > 4) (Figure 7). The result indicated that the relative abundance of *Bacteroides*, *Bacteroidaceae*, *Bacteroides_barnesiae*, *Bacteroides_caecigallinarum* were higher in the HPU group, while the relative abundances of *Negativicutes*, *Lactobacillaceae*, and *Lactobacillales* were significantly enriched in the CON group.

### 3.9. Correlation Analysis of Uterine Microbiota (Genus Level)

**Figure 8:**
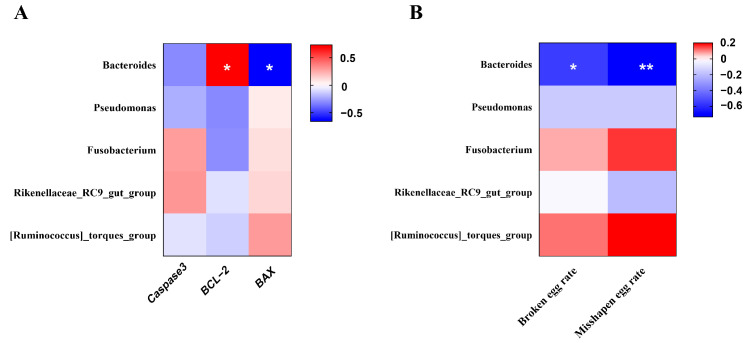
Heatmap of the correlation analysis. (**A**) between uterine microbiota (at genus level) and cell apoptosis-related gene expression. (**B**) between uterine microbiota (at genus level) and unqualified eggs. The red and blue matrices, respectively, represent positive and negative correlations. The color intensity shows Spearman’s r-value of correlation in each matrix. The asterisk indicates a significant correlation (* *p* < 0.05; ** *p* < 0.01).

Spearman’s rank correlation analysis was performed to explore the potential relationship between uterine microbiota (genus level) and the mRNA expression of apoptosis-related genes in the uterus (Figure 8A). In addition, correlation analysis was also performed between uterine microbiota (genus level) and the broken egg rate or misshapen egg rate (Figure 8B). *Bacteroides* showed a significant negative correlation with the *BAX* mRNA expression and a significant positive correlation with the *BCL-2* mRNA expression (*p* < 0.05). Interestingly, *Bacteroides* was also negatively correlated with the broken egg rate and misshapen egg rate (*p* < 0.05).

## 4. Discussion

Eggshells require adequate thickness and hardness to protect embryos from external environmental harm and supply calcium during chick embryo development [27]. With aging, the gradual decline in reproductive organ physiology associated with uterine aging results in decreased eggshell thickness. Uterine aging has been identified as a key factor for the deterioration of eggshell quality in hens [28]. Previously, increasing dietary calcium for aged laying hens was once considered an effective way to improve eggshell quality [29]. While recent studies have shown that high calcium supplementation has a limited effect on improving shell quality, non-nutritive additives like phytoflavonoids are quite an effective strategy [18]. Consistent with this, dietary puerarin supplementation significantly improved eggshell quality and reduced broken eggs. In this study, dietary puerarin supplementation improved uterine function in aged breeder hens, as evidenced by enhanced calcium transport and alleviated histological damage. Our results highlight the importance of improving eggshell quality through non-nutritive feed additives programming in aged broiler breeder hens.

For poultry, dietary calcium is primarily absorbed through the duodenum, which is critical for maintaining systemic calcium homeostasis [30]. With aging, the structure and function in the duodenum undergo a decline, reducing nutrient absorption capacity (including calcium and phosphorus) [31]. Li et al. [32] revealed that puerarin glycoside significantly increased blood calcium and phosphorus in mice. Similarly, dietary puerarin significantly increased serum calcium and phosphorus in aged hens. Herein, we surmise that puerarin protects against age-impaired intestinal calcium absorption in aged hens. Ca^2+^-ATP enzymes are key regulators of calcium balance and transport, which are mainly found in the intestine and uterus [33]. In this study, we found that dietary puerarin improved the activity of Ca^2+^-ATPase in the duodenum, agreeing with increased serum calcium. Additionally, a previous study confirmed that increased serum calcium concentration is beneficial for improving eggshell quality [34]. Thus, improved serum calcium may be one of the reasons for the increased eggshell strength. However, the specific mechanisms through which puerarin influences intestinal calcium absorption remain to be clarified.

Throughout egg formation, eggshell mineralization occurs in the uterus, where uterine epithelial cells absorb calcium ions from the blood and are transported to the uterine cavity, interacting with bicarbonate ions to form calcium carbonate, facilitated by an organic matrix [35]. Thus, intact and healthy uterine tissue, along with genes related to the calcium transportation pathway highly expressed, are the prerequisites for promoting eggshell calcification [18]. Uterine aging has been identified as a key factor in the deterioration of eggshell quality in hens. Our previous study found that uterine epithelial thickness and gland density are critical for eggshell formation [2]. Dietary plant flavonoids improve the proliferation of uterine epithelial cells in aged hens, essential for eggshell calcification [2]. A previous study has shown that puerarin could activate estrogen receptors, including *ERα* and *ERβ*, promoting cellular proliferation [36]. Consistently, puerarin improved uterine morphology (including increasing uterine epithelial thickness) and upregulated *ERα* mRNA expression. This may be due to puerarin possessing a positive estrogen effect in aged hens and promoting uterine cell proliferation. But it still needs further verification. In addition, calcium storage, mobilization, and transport can also be regulated by estrogen receptor-mediated [33]. In general terms, dysregulated gene expression involved in eggshell formation in the uterus correlates with reduced eggshell quality. Calbindin-D 28K (*CABP-28K*), the only calcium-binding protein that exists in chickens, facilitates intestinal calcium transport and is transcriptionally regulated by the estrogen receptor (ER) signaling pathway [9]. As a major phosphorylated protein in the eggshell matrix, Osteopontin (*OPN*) is essential for regulating eggshell calcification [37]. In this study, dietary puerarin significantly increased the transcription levels of *CABP-28K*, *OPN*, and *KCNA1*. Increased serum calcium concentration and enhanced Ca^2+^-ATPase activity indicate that dietary puerarin supplementation promotes uterine calcium transport, thereby improving eggshell quality. The above results demonstrate that puerarin enhances eggshell thickness by activating the estrogen signaling pathway and upregulating the expression of calcification-related genes.

A widely accepted hypothesis posits that aging-induced organ dysfunction stems from accumulated oxidative damage in tissues [38]. Excessive reactive oxygen species (ROS) cause oxidative stress, while antioxidant and free radical scavenger supplementation mitigates aging [17,19,39]. Glutathione peroxidase (GSH-Px) and superoxide dismutase (SOD) are key antioxidant enzymes in cells to eliminate ROS [40]. Furthermore, total antioxidant capacity (T-AOC) and malondialdehyde (MDA) are indicators for evaluating the balance between oxidants and antioxidant factors [41]. Recent studies have found that puerarin treatment markedly altered SOD, MDA, and T-AOC contents in the serum of S. enterica-infected chicks [42]. Du et al. confirmed that 200 to 800 mg/kg puerarin enhanced the serum and intestinal antioxidant capacity in post-peak laying hens [22]. Consistent with these findings that dietary puerarin increased uterine T-AOC and GSH-Px while reducing MDA levels in aged hens. The nuclear transcription factor 2 (*Nrf2*), a key transcription factor, activates oxidative defense systems [43] by upregulating antioxidant enzymes via antioxidant response elements. Recent studies have found that puerarin induced antioxidant defense by activating the *Nrf2*/*HO-1* signal pathway to protect against oxidative stress [44,45]. In a similar vein, we found that dietary PU increased mRNA expression of *Nrf2* and *HO-1* in the uterine. This indicates that puerarin could alleviate uterine oxidative damage via activating the *Nrf2*/*HO-1* pathway. Furthermore, excessive oxidative damage can damage intracellular components, leading to apoptosis. Caspases, a group of essential proteases, are activated during apoptosis and play a pivotal role in both the initiation and execution of the apoptotic process [46]. Amongst these, *Caspase 3* is a key executor of apoptosis. Members of the *BCL-2* family, including *BCL-2* and *BAX*, are central regulators of apoptosis that promote (*BAX*) or inhibit (*BCL-2*) cell death [47]. Therefore, the *BAX*/*BCL-2* ratio is an important indicator of apoptosis, and a higher *Bax*/*Bcl-2* ratio indicates a stronger pro-apoptotic capacity [2]. In our experiment, puerarin reduced *Caspases 3* expression and the *BAX*/*BCL-2* ratio, indicating its potential to attenuate aging-induced uterine apoptosis. Meanwhile, the TUNEL assay also demonstrated that puerarin treatment decreased glandular cell apoptosis. In the present study, we also observed that the distribution of tubular gland cells was irregular in aged hens. The tubular gland cells of the shell gland are the major source of eggshell calcium [9]. With aging, decreased shell gland density could directly affect calcium transport and reduce the quality of the eggshell. Here, puerarin alleviated the morphological damage and glandular apoptosis, which were believed to have close links with enhancing the calcium transport in aged hens.

It is well-established that diverse microorganisms reside in the intestines and reproductive tracts of animals, forming symbiotic relationships with their hosts. These microbial communities significantly influence the host’s physiology, health, and metabolism [48]. The species and number of microbes normally stay balanced within the bodies of animals. However, with aging, the microbial flora undergoes apparent changes, leading to an imbalance in microbiota abundance compared to younger ages [49]. Recent research has shown that uterine microbiota dysbiosis exacerbates aging processes through disruption of cell programming related to the cell cycle, proliferation, and apoptosis in aged versus young hens [15]. Notably, their results revealed a higher abundance of *Actinobacteria* and a lower abundance of *Bacteroidota* in the aged uterus. In this study, we found that dietary puerarin reversed trends of change in abundance of *Actinobacteria* and *Bacteroidota* induced by aging. Furthermore, the gut microbiota diversity in the uterus was significantly altered after puerarin intervention. The change in microbial abundance suggests that it may correlate with some of the physiological function improvements in the uterus in physiological aging. For poultry, the cloaca connects reproductive and digestive tracts, enabling gut–oviduct microbial exchange [15]. There is evidence that intestinal microbes can be transmitted to the reproductive tract and colonize, altering its ecosystem [50]. *Bacteroides* is a dominant genus within the intestinal microbiota and plays a vital role in modulating host immune and intestinal functions [51]. Here, we found a significant increase in *Bacteroides* in the uterus. Analogously, the correlation analysis showed that the abundance of *Bacteroides* had a highly negative correlation with pro-apoptotic *BAX*. Hence, the increased abundance of Bacteroides might contribute to the alleviation of pathological changes and metabolic function in the uterus of aged hens following puerarin supplementation. This may be related to age-associated changes in gut *Bacteroides* abundance, but further research is required to confirm this. The findings of Lu et al. show that probiotics improve eggshell quality via regulating microbial composition in the uterine and cecum [16]. Of those, *Bacteroides* demonstrated positive correlations with cholesterol, effective thickness, and concentrations of ions in eggshell. Our results are consistent with their findings; the abundance of *Bacteroides* had a highly negative correlation with the broken egg rate and misshapen egg rate, indicating that uterine microbiota influence eggshell formation. In summary, the change in the microbial community composition in the uterus induced by dietary puerarin is one of the potential causes to improve the uterine physiological function and eggshell quality in aged hens. Future research needs to focus on the role of microbes in uterine microenvironments that can utilize more targeted design strategies for improving aging-related physiological changes.

## 5. Conclusions

In conclusion, dietary puerarin supplementation reduces cell apoptosis and enhances the antioxidant capacity by activating the *Nrf2*/*HO-1* pathway, thereby alleviating aging-induced uterine injury. Further, puerarin modulates uterine microbiota, improves uterine morphology, and upregulates calcium transport-related gene expression, ultimately enhancing eggshell quality. Consequently, puerarin has the potential to serve as a functional feed additive in aged breeder hens to improve uterine health and function.

## Figures and Tables

**Table 1:** Composition and nutrient levels of the basic corn–soybean diet.

Ingredient	Content (%)	Nutrient Level ^2^	Value
Corn	62.48	Avian metabolic energy (MJ·kg^−1^)	11.48
Soybean meal	23.40	Crude protein (%)	15.94
Limestone	8.70	Available phosphorus (%)	0.51
Soybean oil	2.10	Methionine (%)	0.49
Dicalcium phosphate	2.01	Lys (%)	0.80
NaCl	0.35	Thr (%)	0.58
Mineral premix ^1^	0.62		
Choline chloride (50%)	0.12		
DL-Methionine	0.22		
Total	100.00		

^1^ Mineral premix includes: vitamin A, 12,500 IU; vitamin D3, 2500 IU; vitamin K3, vitamin E, 30 mg; 2.60 mg; thiamine, vitamin B12, 0.025 mg; 2 mg; folic acid, 1.25 mg; riboflavin, 6 mg; biotin, 0.0325 mg; pantothenic acid, 12 mg; niacin, Mn, 100 mg; 50 mg Cu, 8 mg; Fe, 80 mg; Zn, 75 mg; Se, 0.2 mg; I, 0.35 mg (per kg complete diet). ^2^ Nutrient levels were calculated values.

**Table 2:** Effect of dietary puerarin supplementation on broken egg rate and misshapen egg rate of aged hens.

Items	CON	LPU	HPU	*p*-Value
Broken egg rate (%)				
weeks 1 to 4	2.93 ± 0.36	1.95 ± 0.46	2.26 ± 0.45	0.273
weeks 5 to 8	3.16 ± 0.46 ^a^	2.94 ± 0.36 ^a,b^	1.70 ± 0.18 ^b^	0.024
weeks 1 to 8	3.04 ± 0.37 ^a^	2.45 ± 0.25 ^a,b^	1.98 ± 0.31 ^b^	0.006
Misshapen egg rate (%)				
weeks 1 to 4	1.75 ± 0.38	1.11 ± 0.22	1.29 ± 0.78	0.285
weeks 5 to 8	1.81 ± 1.21	1.36 ± 0.78	1.02 ± 0.24	0.161
weeks 1 to 8	1.78 ± 1.22	1.24 ± 0.88	1.16 ± 0.21	0.084

Mean value ± SEM is used to represent data (n = 8). ^a, b^ Different letters represent statistically significant differences among the groups (*p* < 0.05).

**Table 3:** Effect of dietary puerarin supplementation on eggshell quality of aged hens.

Items	CON	LPU	HPU	*p*-Value
4 weeks				
Shape index	1.28 ± 0.02	1.30 ± 0.01	1.31 ± 0.01	0.599
Eggshell weight (g)	5.93 ± 0.17	6.05 ± 0.15	6.08 ± 0.11	0.754
Shell strength (kg/cm^2^)	3.30 ± 0.14	3.75 ± 0.14	3.85 ± 0.10	0.053
Shell thickness (mm)	0.36 ± 0.03	0.38 ± 0.02	0.39 ± 0.03	0.094
8 weeks				
Shape index	1.33 ± 0.02	1.33 ± 0.02	1.38 ± 0.02	0.396
Eggshell weight (g)	5.94 ± 0.16	5.76 ± 0.11	6.11 ± 0.12	0.195
Shell strength (kg/cm^2^)	2.84 ± 0.18	3.17 ± 0.11	3.38 ± 0.22	0.078
Shell thickness (mm)	0.29 ± 0.01 ^a^	0.30 ± 0.01 ^a,b^	0.31 ± 0.02 ^a^	0.012

Mean value ± SEM is used to represent data (n = 16). ^a, b^ Different letters represent statistically significant differences among the groups (*p* < 0.05).

**Table 4:** Effect of dietary puerarin supplementation on the relative abundance of bacteria at the phylum and genus levels in the uterus.

Items	CON	HPU	*p*-Value
Phylum			
*Bacteroidota*	31.62 ± 7.91	49.73 ± 3.94	0.056
*Firmicutes*	44.75 ± 3.94	36.62 ± 2.02	0.864
*Proteobacteria*	8.91 ± 5.74	3.70 ± 0.63	0.323
*Fusobacteriota*	5.35 ± 3.30	1.34 ± 0.35	0.292
*Actinobacteriota*	1.70 ± 0.48	0.69 ± 0.10	0.071
Genus			
*Bacteroides*	10.88 ± 2.23	23.93 ± 2.72 *	0.007
*Pseudomonas*	4.55 ± 3.91	1.12 ± 0.48	0.410
*Fusobacterium*	5.35 ± 3.30	1.34 ± 0.25	0.260
*Rikenellaceae_RC9_gut_group*	8.15 ± 2.57	10.10 ± 1.34	0.520
*[Ruminococcus]_torques_group*	4.20 ± 0.70	3.41 ± 0.42	0.157

Mean value ± SEM is used to represent data (n = 5). Asterisks indicate significant differences between groups (*p* < 0.05).

## Data Availability

The data that support the findings of this study are available from the corresponding author upon reasonable request.

## Supplementary Materials

The following supporting information can be downloaded at: https://www.mdpi.com/article/10.3390/antiox14080960/s1, Table S1: Primer sequences used in the present study.

## Abbreviations

The following abbreviations are used in this manuscript:

*CaBP-28K*	The calcium-binding protein calbindin-28K
*Nrf2*	Nuclear factor-erythroid 2-related factor 2
*HO-1*	Heme oxygenase-1
*CAT*	Catalase
*GPX1*	Glutathione peroxidase 1
*SOD1*	Superoxide dismutase-1
*Caspase3*	Cysteine-aspartic protease 3
*BAX*	BCL2-associated X protein
*BCL-2*	B-cell lymphoma 2
*ERα*	Estrogen receptor α
*ERβ*	Estrogen receptor β
*OPN*	Osteopontin
*SYT15*	Synaptotagmin 15
*CDH6*	Cadherin 6
*KCNA1*	Voltage-gated potassium channel subfamily A member 1
OTUs	Operational Taxo-nomic Units
LEfSe	LDA Effect Size analysis
