# Supplementary material for: Puerarin Enhances Eggshell Quality by Mitigating Uterine Senescence in Late-Phase Laying Breeder Hens
Source: Antioxidants (Basel). 2025 Aug 5;14(8):960. doi: 10.3390/antiox14080960 (PMC12382635; doi:10.3390/antiox14080960)
Supplement: Supplementary file 1 [file antioxidants-14-00960-s001.zip › antioxidants-3782048-supplementary.pdf]

Table S1 Primer sequences used in the present study.

Gene	GeneBank ID	Primer sequence (5′→ 3′)	Product size (bp)
ACTB	NM_205518.1	F: TGTTACCAACACCCACACCC<br>R: TCCTGAGTCAAGCGCCAAAA	110
Nrf2	NM_205117.1	F: GATGTCACCCTGCCCTTAG<br>R: CTGCCACCATGTTATTCC	216
Keap1	XM_025145847.1	F: CAGCGTGAGAGGTGAGTATGAG<br>R: CTGTTGAGCTGATCCGTCC	289
HO-1	NM_205344.1	F: GCGGAGAACACACCCTTCAT<br>R: GGATCTCTGCCCTCCAGTTG	235
CAT	NM_001031215.2	F: TGCTGGCAACCCAATAGGAG<br>R: AATAGCCAAAGGCACCTGCT	164
GPX1	NM_001105664.2	F: TCACCATGTTCGAGAAGTGC<br>R: ATGTACTGCGGGTTGGTCAT	207
SOD1	NM_205064.1	F: TTGTCTGATGGAGATCATGGCTTC<br>R: TGCTTGCCTTCAGGATTAAAGTGAG	98
CABP-28K	NM_205513.1	F: CCTCAGGTGAACTCAGGCAA<br>R: AACATGCCAAGACCAAGGCT	84
SYT15	NM_205079.1	F: GCATCCGAAAGGCTCCTCAT<br>R: GCATCTTTCATCAGGCAGCAG	108
OPN	NC_006091.5	F: TCAGCAAGGTCAAACACCCC<br>R: GTCAGATGTCTGCAGGGTGA	229
KCNA1	XM_004938075.3	F: TGCGGTACTTCGACCCTTTG<br>R: GCTGGTATTCTCCCTCTGGC	241
CDH6	NM_001001758.2	F: AACGAGTCGGGCTACTACCT<br>R: GATTCCTCAGCGTGCCGATA	245
ERα	NM_205183.2	F: TGTGCTGTGTGCAACGACTA<br>R: CAGGCCTGGCAACTCTTTCT	167
ERβ	NM_204794.2	F: CGGGCGTGGTGACATTAAAC<br>R: CCAGGATGAAGGGTGTGCAA	192
Caspase3	NM_204725.2	F: TGTCTGTCATCATGGCTCTTG<br>R: CAACGGAGGATGGGATGCCT	183
BCL-2	NM_205339.2	F: CAACGGAGGATGGGATGCCT<br>R: AAGCGCCAAGAGTGATGCAA	131
BAX	XM_001235092.4	F: TCCATTCAGGTTCTCTTGACC<br>R: GCCAAACATCCAAACACAGA	119
PCNA	NM_204170	F: GGCGTCAACCTAAACAGCAT<br>R: GCTCCACATCGAGGTCCATA	169

F: represents forward, R: represents reverse.
